# Urogenital Schistosomiasis in Fisherman, Nepal, 2019

**DOI:** 10.3201/eid2607.191828

**Published:** 2020-07

**Authors:** Ranjit Sah, Jürg Utzinger, Andreas Neumayr

**Affiliations:** Tribhuvan University Teaching Hospital, Kathmandu, Nepal (R. Sah);; Swiss Tropical and Public Health Institute, Basel, Switzerland (J. Utzinger, A. Neumayr);; University of Basel, Basel (J. Utzinger, A. Neumayr)

**Keywords:** schistosomiasis, urogenital, Nepal, *Schistosoma* *haematobium*, bilharziosis, zoonoses, trematodes, parasites

## Abstract

We report a case of urogenital schistosomiasis in a 34-year-old male patient in Nepal and summarize additional case reports. These cases provide putative evidence for the potential existence of human-pathogenic (most likely zoonotic) schistosome species on the Indian subcontinent.

We report the case of a 34-year-old male patient from Siraha District, Nepal, in the outer Terai Region bordering India. The patient was referred to us in October 2019 by his regional hospital because of a diagnosed microhematuria and reported intermittently observed episodes of macrohematuria. The patient reported no relevant medical history and had never traveled abroad other than to visit neighboring districts in Nepal and across the border to India. He earned his living as a fisherman.

Results of the physical examination were unremarkable. Laboratory test results showed a white cell count within reference ranges with mild eosinophilia (14%; 728 cells/µL) and unremarkable results for renal and liver function tests. Urine analysis confirmed microhematuria, and microscopic examination of the urine sediment showed very few but typical trematode eggs, resembling those of *Schistosoma haematobium*, the causative agent of urogenital schistosomiasis (*1*) (Figure, panel A). An abdominal ultrasound examination revealed diffuse bladder wall thickening, which was confirmed by computed tomography (Figure, panel B).

**Figure:**
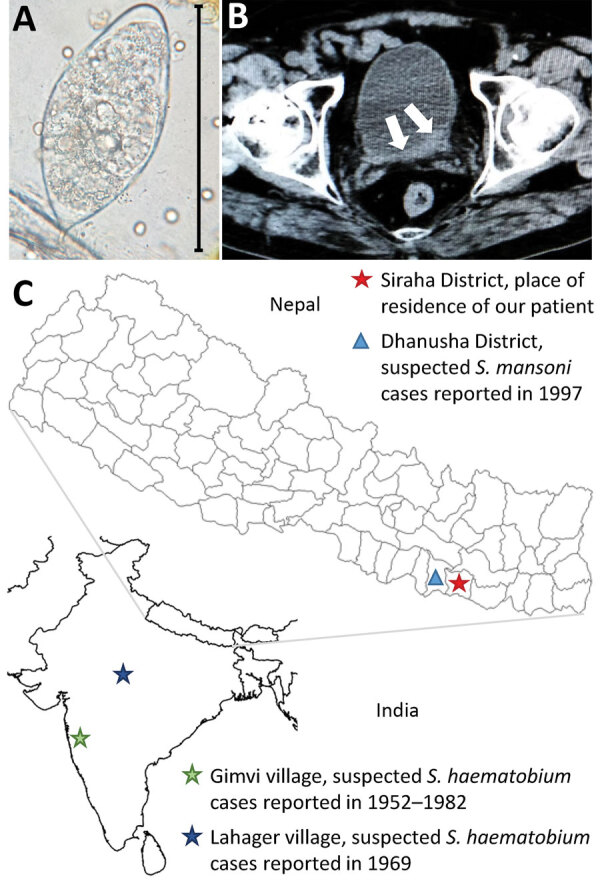
Investigation of urogenital schistosomiasis in a 34-year-old male patient in Nepal. A) Trematode egg, resembling the typical *Schistosoma haematobium* morphology, detected upon microscopic investigation of patient’s urine sediment. Original magnification ×40. B) Computed tomography image showing bladder wall thickening (white arrows). C) Geographic location of patient’s place of residence and of cases of human schistosomiasis reported previously in India and Nepal.

After praziquantel treatment (40 mg/kg for 3 d), hematuria resolved, and no more eggs were detectable at follow-up 2 weeks later.

Although several zoonotic schistosome species have been reported from the Indian subcontinent, human-pathogenic schistosome species are considered absent from the region (*2*) because none of the known intermediate host snails involved in the lifecycle of human-pathogenic schistosome species are present. Nevertheless, some reports of parasitologic confirmed autochthonously acquired infections question the assumption that human schistosomiasis is nonexistent on the subcontinent.

In 1952, Gadgil and Shah (*3*) reported the detection of terminal-spined trematode eggs resembling those of *S. haematobium* in human urine samples from Gimvi, a village 250 km south of Bombay in India. In Gimvi village, terminal-spined trematode eggs were detected in 250 of 1,200 villagers; hematuria was the most common clinical manifestation. At the time of this discovery, available information suggested that the infectious focus area had existed for at least 60 years (*3*). Malacologic investigations in the river running through the village and infection experiments identified the *Ferrissia tenuis* snail as a competent intermediate host that, to date, has been reported nowhere else as intermediate host of *S. haematobium* (*4*). After the river was treated with copper sulfate and the patients with antimontartrate, a follow-up survey in 1957 showed that 11.1% of the male and 8.7% of the female villagers were still infected; the highest infection rate (36.8%) was observed in boys 10–15 years of age (*5*). After the introduction of praziquantel in the 1980s, the Gimvi focus was successfully eliminated (*6*).

The parasite identified at Gimvi village was named and reported as *S. haematobium* on the basis of its egg morphology and the clinical manifestation in infected patients. However, the lifecycle of the Gimvi schistosome does not include the classical snail intermediate host genus *Bulinus,* raising questions about on its identification as *S. haematobium* in the 1950s. Unfortunately, parasitologic samples from that time are no longer available for in-depth molecular testing.

In 1969, seventeen years after the first description of the Gimvi cases, Shrivastava and Arora (*7*) reported the finding of *Schistosoma* typical egg granuloma in the bladder biopsy of a 26-year-old woman from Lahager village, situated in Raipur District of Madhya Pradesh, India. A village survey revealed a high prevalence of hematuria among the villagers. Upon urine microscopy, eggs “highly suspicious of *S. haematobium*” were found in some of the samples and “in one deposit features of *S. haematobium* eggs were seen.” No further cases were reported from this region thereafter.

In 1997, Sherchand and Ohara reported the finding of lateral-spined trematode eggs, resembling those of *S. mansoni*, in a fecal sample from an inhabitant of Dhanusha District in the Terai Region, Nepal, during a coprologic survey conducted in 1995 (*8*). In 1997, similarly shaped eggs were detected in fecal samples of another 2 inhabitants in Dhanusha District (*9*). No clinical data are available for these 3 cases. In 1999, a serologic survey conducted among 508 inhabitants of the same district, reported an overall seroprevalence of 18.1% (range 1%–42.7%, depending on the surveyed village) (*9*). Of interest, Dhanusha is the neighboring district to that of the patient we report.

In summary, it appears that *Schistosoma* species capable of infecting humans are present on the Indian subcontinent. However, although the reported morphology of microscopically detected eggs in fecal samples resemble those of human-pathogenic species, the local absence of any classical intermediate host snails and the apparently low number of cases questions the assumption that endemic foci may simply have been overlooked in the past. We suspect that our case, as well as the previously reported cases, most likely depicts zoonotic infections. Unfortunately, we were unable to continue follow-up on the patient we describe, and no diagnostic samples were stored that would allow us to clarify the species by molecular-genetic investigations. We hope this shortfall can be rectified in future cases.
